# Epidemiological methods in transition: Minimizing biases in classical and digital approaches

**DOI:** 10.1371/journal.pdig.0000670

**Published:** 2025-01-13

**Authors:** Sara Mesquita, Lília Perfeito, Daniela Paolotti, Joana Gonçalves-Sá

**Affiliations:** 1 Social Physics and Complexity (SPAC) Lab, LIP–Laboratory for Instrumentation and Experimental Particle Physics, Lisboa, Portugal; 2 Nova Medical School, Lisboa, Portugal; 3 ISI Foundation, Turin, Italy; 4 Nova School of Business and Economics, Carcavelos, Portugal; Instituto Politécnico Nacional Escuela Superior de Medicina: Instituto Politecnico Nacional Escuela Superior de Medicina, MEXICO

## Abstract

Epidemiology and Public Health have increasingly relied on structured and unstructured data, collected inside and outside of typical health systems, to study, identify, and mitigate diseases at the population level. Focusing on infectious diseases, we review the state of Digital Epidemiology at the beginning of 2020 and how it changed after the COVID-19 pandemic, in both nature and breadth. We argue that Epidemiology’s progressive use of data generated outside of clinical and public health systems creates several technical challenges, particularly in carrying specific biases that are almost impossible to correct for *a priori*. Using a statistical perspective, we discuss how a definition of Digital Epidemiology that emphasizes “data-type” instead of “data-source,” may be more operationally useful, by clarifying key methodological differences and gaps. Therefore, we briefly describe some of the possible biases arising from varied collection methods and sources, and offer some recommendations to better explore the potential of Digital Epidemiology, particularly on how to help reduce inequity.

## Introduction

Epidemiology is the study of health patterns and determinants in populations [1]. It relies on diverse health-related data from various sources, including questionnaires, laboratory tests, and sociodemographic information. In recent years, Digital Epidemiology, a subfield of Epidemiology, has emerged, driven by the widespread adoption of digital technology and computational power [2]. Initially, Digital Epidemiology was defined as the application of digital data sources, such as mobile phone records, social media, and other digital platforms, to monitor and analyze health and disease patterns in populations [3]. This definition was later refined to include both a broad and a narrow perspective: broadly, Digital Epidemiology is epidemiology that uses digital data, and narrowly, it is the use of digital data generated outside the public health systems, particularly data not originally intended for epidemiological purposes [4]. These definitions emphasize the source (outside/inside of health systems) and the format (digital) of the data.

However, (1) data sets are now almost universally digital, encompassing clinical, social network, and classical field survey data; (2) epidemiology has a long history of re-purposing data sets beyond those collected solely for epidemiological studies, including data related to public housing, human and animal density, traffic, weather, and postal codes; and (3) epidemiology increasingly relies on nontraditional yet clinical data sets, such as electronic medical records, prescription records, and on-call triage systems for purposes like disease incidence estimation and syndromic surveillance. For example, the historical Oxford Record Linkage Study, utilized routine hospital records for epidemiological research [5], and illustrates how traditional epidemiology has long integrated secondary data with rigorous planning.

**Fig 1:**
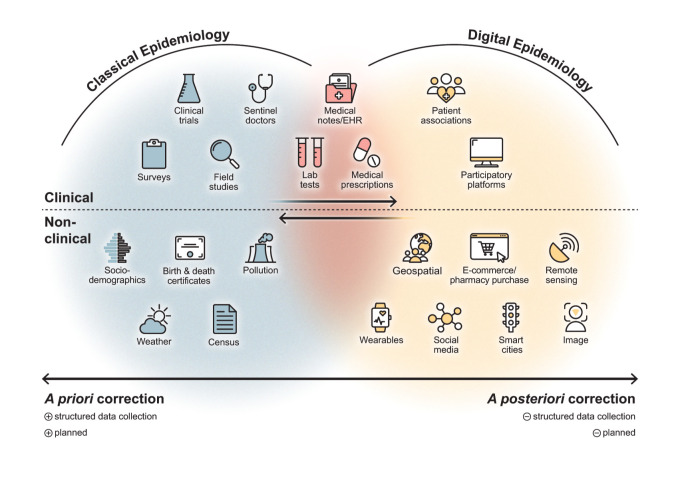
Contrasting *a priori* and *a posteriori* bias correction in Classical vs. Digital Epidemiology. Classical Epidemiology data is typically more structured, with planned study designs leading to higher quality but slower, resource-intensive data collection and less frequent updates. In contrast, Digital Epidemiology can offer near real-time findings, greater reach, and community engagement but faces challenges with data privacy, standardization, and quality variability, along with risks of digital divides. Certain data sources, such as weather, pollution, or sociodemographics, can fit both categories but differ in their collection methods—structured under institutional supervision in Classical Epidemiology versus more unstructured, secondary collection in Digital Epidemiology. Despite their differences, both Classical Epidemiology and Digital Epidemiology can validate and improve each other. As data digitization progresses, the distinction between Classical and Digital Epidemiology blurs, emphasizing the need for a comprehensive and evolving definition of Digital Epidemiology. In the figure, the arrows illustrate this ongoing integration and the terms *a priori* and *a posteriori* refer to the timing of bias correction in relation to data collection.

One key difference is on how biases are are addressed and, in this context, bias refers to a systematic error in the design, conduct, or analysis of a study, leading to consistently inaccurate results that deviate from the true values in the population [6]. In Classical Epidemiology, studies are carefully planned with a public health goal in mind, focusing on statistical soundness through structured data collection. For example, in clinical trials, potential biases—such as selection bias, small sample sizes, and confounding factors—are minimized *a priori* by design. Even secondary data, such as weather or census records, is often collected with statistical rigor or in structured formats. In contrast, Digital Epidemiology often relies on secondary data, collected from digital platforms or apps (Fig 1), that was generated without public health goals, nor concerns of representativeness and generalizability, making biases more likely to be identified *a posteriori*. While *a posteriori* methods are also employed in Classical Epidemiology to correct biases after data collection, they typically serve as corrective measures rather than primary strategies. Conversely, some *a priori* methods—such as optimizing user engagement or reducing dropout—are sometimes possible in Digital Epidemiology but limited to the few situations during which researchers have some control over the data collection. Additionally, Digital Epidemiology data sets frequently tap into different (sub-)populations, and may be collected without explicit consent, presenting new ethical and methodological challenges. We draw from this methodological difference, to propose an updated definition of Digital Epidemiology, both conceptual and operational: it builds on the existing definitions but removes focus from the (digital) nature of the data or its sources, emphasizing instead the issues posed by the statistical nature of secondary data. Defining Digital Epidemiology by “the use of digital data that was not originally collected with epidemiological statistical rigor,” stresses two of its main challenges: (1) applying this repurposed data effectively in epidemiological research, while addressing the inherent biases introduced during such data collection and processing; (2) developing new ethical and privacy protecting methodologies that effectively integrate both classical and digital approaches, ultimately contributing to improved public health outcomes.

In this paper, we focus on infectious diseases as these account for over 50% of Digital Epidemiology articles [7], and their surveillance typically relied on laborious, costly, and slow processes, such as those conducted by sentinel doctors, surveyors, and testing labs [8]. We begin with a summary of early 2020’s Digital Epidemiology of infectious diseases and describe how the COVID-19 pandemic led to significant transformations in the field [9]. We argue that the pandemic helped not only to reveal how traditional systems can be complemented (e.g., by utilizing diverse data streams, enabling the rapid identification of disease dynamics, risk factors, and optimized response strategies [10]), but also why the emphasis on statistical bias is crucial.

We then highlight the differences between Classical and Digital Epidemiology in terms of data, methodologies, and approaches to bias mitigation (Table 1) and provide recommendations to enhance Epidemiology’s evolving role, acknowledging its statistical and ethical challenges.

**Table 1:** Biases and mitigation strategies in Classical and Digital Epidemiology.

Bias	Classical	Digital	Key Differences
**1. Sampling and Representation**			
Selection and Coverage *Selection bias arises from non-random sampling or selective participation*, *while coverage bias*, *a subset of selection bias*, *occurs due to incomplete coverage of the target population in the sampling frame*. *Both can result in over- or underrepresentation of certain groups* [74–77].	Due to the selection process or coverage limitations of the data source. Clinic-based studies often include individuals seeking care, underrepresenting healthier people or people with no healthcare access. **Mitigation:** *A priori*—Use random and stratified sampling; expand the sampling frame. *A posteriori*—Apply statistical adjustments; combine data sets; use self-reported outcomes.	Self-selection in online surveys or apps often overrepresents tech-savvy, younger individuals and underrepresents older, less connected people (not using digital platforms or with limited internet access). **Mitigation:** *A priori*—Analyze random samples (e.g., on social networks) instead of relying on keyword searches; recruit cohort or user panels. *A posteriori*—Apply data weighting; integrate diverse sources; promote digital literacy; conduct audits; ensure ethical reviews and “opt-in” methods, to build trust; maintain transparency; engage stakeholders.	Classical relies on structured population sampling, while Digital Epidemiology must deal with self-selection and coverage gaps due to digital divides (disparities in access to digital technologies).
Detection and Surveillance *Results from differences in diagnostic methods or frequency*, *affecting exposure-outcome associations*. *Surveillance bias occurs with intensified monitoring (e*.*g*., *frequent screenings for certain patient groups)*, *leading to overestimated associations* [6,77–80].	Different methods or frequencies used across groups, leading to overestimated associations (e.g., patients on specific medications undergoing more frequent screenings). **Mitigation:** *A priori*—Standardize diagnostic criteria and protocols; hide exposure status from researchers. *A posteriori*—Use statistical adjustments; stratify by disease severity; control for visit frequency.	Inconsistencies occur due to varying data intensity across digital platforms (e.g., wearables, social media), resulting in more frequent detection among heavy tech users. **Mitigation:** *A priori*—Promote equal access to technology; use patient groups to increase samples. *A posteriori*—Apply statistical normalization, cross-validate with independent data sets, use inverse probability weighting, apply multiple imputation, and perform sensitivity analysis to account for detection inconsistencies. Integrate traditional and digital data sources to reduce bias.	Classical Epidemiology relies on standardized protocols, ensuring consistent detection but often has slower access to data. Digital Epidemiology can rapidly access real-time data, especially from less-surveyed populations through opt-in platforms, social networks, and patient groups, but it faces greater variability in detection intensity and less control over health condition identification.
**2. Measurement and Information**			
Measurement *Systematic errors in data collection*, *labeling*, *or measurement*, *leading to inaccurate or inconsistent information*. *This bias can arise from faulty instruments*, *differences in measurement techniques*, *or inaccurate self-reporting* [80].	Using different devices for measuring (e.g., blood pressure) yields varying results; disparity in COVID-19 data quality across regions. **Mitigation:** *A priori*—Instrument calibration; standardize measurement tools and protocols; train personnel consistently; use objective data collection methods. *A posteriori*—Apply statistical corrections; perform sensitivity analysis; validate with external data sources.	Device inaccuracies (e.g., inconsistent readings from wearables); varying accuracy in self-reported data from apps or other platforms. **Mitigation:** *A priori*—Calibrate digital devices; establish data labeling guidelines. *A posteriori*—Cross-validate with other sources; data cleaning; use multiple imputation, regression calibration, or inverse probability weighting. Machine learning methods (e.g., Random Forests, Gradient Boosting) can be applied cautiously.	Classical Epidemiology has more controlled and standardized measurement processes, but may still encounter inaccuracies. Digital Epidemiology, while offering vast data collection, often faces greater challenges in maintaining measurement consistency due to device variability and user-generated data.
Information and Recall *Both relate to inaccuracies in how data is collected or reported*. *Information bias arises from measurement errors or misclassification of exposure or disease status; recall bias occurs when participants inaccurately remember or report past events* [6,77–79].	Uncalibrated devices or inconsistent interviewer techniques cause misclassification; participants inaccurately remembering past exposures or symptoms, particularly in retrospective studies. **Mitigation:** *A priori*—Use validated instruments; standardize data collection protocols; train interviewers. *A posteriori*—Apply statistical corrections; validate with independent sources (e.g., medical records).	Inaccuracies in self-reported data (e.g., symptoms on social media) due to social desirability or incorrect reporting. **Mitigation:** *A priori*—Use validated digital tools; offer information to users on how to use them. *A posteriori*—Data cleaning (e.g., NLP); cross-check self-reported with passive data (e.g., location tracking).	Classical methods rely on standardized tools, minimizing measurement errors but remain prone to recall inaccuracies. Digital methods, being real-time, reduce recall bias as data is captured instantly, but face greater variability in information accuracy due to inconsistent or unstructured data inputs.
**3. Technological and Platform**			
Availability and Platform *Both biases refer to how data access influences results*. *Availability bias involves selecting subjects or questions based on ease of access to data rather than to clinical relevance*, *while platform bias relates to differences in data collection methods across platforms* [3,81,82].	Using clinical records because they are accessible or preferring “easier” data collection methods (e.g., phone vs. in-person) even when these impact response rates or quality, due to differences in formality or anonymity. **Mitigation:** *A priori*—Conduct literature reviews; use mixed data collection methods (e.g., phone and in-person); prioritize quality collection vs. larger samples. *A posteriori*—Apply weighting techniques; combine data from different sources.	Discrepancies in available data are common and platforms are preferred based on ease and cost (e.g., Twitter vs. Facebook, online vs. clinical paper data, recent vs. older sources). **Mitigation:** *A priori*—Multidisciplinary teams; diverse stakeholders and data sources; standardize data collection (e.g., using unified survey formats). *A posteriori*—Apply demographic weighting; integrate multiple platforms; use correction factors; cross-validate with traditional sources.	While classical approaches can more carefully design mixed data collection to counter platform effects, digital methods must adapt to the inherent variability of online environments and often private and proprietary data sources.
Attrition and Behavioral *Arise from participants dropping out of studies (attrition) or altering responses based on social desirability or judgment (behavioral)*. *Both biases are driven by participants’ decisions and can significantly impact study data quality* [2,83,84].	Underreporting of undesirable behaviors (e.g., smoking) and overreporting of positive behaviors (e.g., exercise). In longitudinal studies, drop out is often linked to key variables that are difficult to control (e.g., age, health status). **Mitigation:** *A priori*—Retention strategies (e.g., regular contact, incentives); survey anonymity; indirect questioning. *A posteriori*—Validate self-reports; intention-to-treat analysis; inverse probability weighting; multiple imputation; sensitivity analyses.	Users can easily stop using a platform (often tied to digital literacy or loss of interest); online behaviors are modulated by strong social desirability; searches or posts may be exaggerated by external events (e.g., pandemics), reflecting anxiety rather than actual behavioral changes. **Mitigation:** *A priori*—Incentive strategies (e.g., offering rewards or gamification elements); use longitudinal data. *A posteriori*—Apply data imputation; combine complementary data sources; use time-series analysis or machine learning to adjust for behavior spikes from external events.	Classical methods have more control over participant follow-up through structured contact, while digital methods must adapt to fluctuating engagement, requiring creative strategies for real-time monitoring and flexible data integration to address unexpected dropouts and behavior shifts.
**4. Causal and Analytical**			
Confounding and Temporal *Affect the ability to establish causal relationships*. *Confounding involves an external factor influencing both exposure and outcome; temporal bias occurs when the timing between exposure and outcome is misjudged* [6,85,86].	Common in observational studies without randomization (e.g., age confounding a drug study as older individuals are both more likely to take that drug and to have higher disease risk). Event order is unclear (e.g., hypertension caused kidney failure or vice versa), especially in retrospective studies with inconsistent time points and limited data due to high costs. **Mitigation:** *A priori*—Randomization, matching, or restriction techniques; hypothesis driven research. *A posteriori*—Multivariate regression, stratification, or propensity score matching; Statistical methods to reconstruct exposure timelines; alignment of data intervals or imputation techniques.	Socioeconomic status can affect both internet/technology use and health outcomes. Temporal bias arises from imprecise timing between exposure and outcome (e.g., health-related posts lacking clear dates or onset timing). **Mitigation:** *A priori*—Prefer platforms with clear time stamps (participatory surveillance); hypothesis-driven research; conduct pilot studies. *A posteriori*—Propensity score matching; integrate multiple data sources. Time-stamped data for temporal bias, infer temporal relationships through algorithms, and validate findings against structured, longitudinal datasets.	Classical Epidemiology uses structured study designs to minimize confounding and temporal biases, while Digital Epidemiology, despite being more prone to these biases from unstructured data, can more easily conduct retrospective and longitudinal studies using large-scale, cost-effective datasets and advanced statistical corrections.
Confirmation and Anchoring *Occurs when researchers selectively interpret or report findings that align with preexisting hypotheses*, *while ignoring contradictory evidence (Confirmation)*. *It also happens when individuals rely heavily on initial*, *often irrelevant*, *information when forming judgments or making decisions*, *leading to skewed conclusions (Anchoring)* [87].	In cohort, case-control studies, or clinical trials, researchers may focus on results that confirm their hypotheses, ignoring neutral or negative findings. Clinicians can overvalue initial information (e.g., first test result or patient history), leading to overconfidence or wrong diagnoses. **Mitigation:** *A priori*—Preregistration of study hypotheses; standardized protocols; blinding researchers. *A posteriori*—External validation with independent data sets; post-stratification; report all findings, including nonsignificant results or contradictory findings.	Researchers may prioritize trends, especially quantitative data that confirm their preconceptions, while ignoring contradictory evidence. Early or real-time data with poorer quality (e.g., early reports, anxiety-related symptoms) may disproportionately influence later interpretations. **Mitigation:** *A priori*—Preregistration of study hypotheses; prespecification of digital data sources and algorithms; cross-platform and interdisciplinary peer review; blinding of researchers. *A posteriori*—Validation with independent datasets; automated inconsistency checks; transparency by publishing all findings; regular analysis updates; ensemble models adjusting with new data.	Classical epidemiology typically follows more structured protocols, but both confirmation and anchoring biases can occur, especially in observational studies. Digital epidemiology faces a greater risk of these biases due to the rapid data influx, the simultaneous testing of multiple hypotheses without making them explicit, and reliance on early digital trends, making it more susceptible to overconfidence in initial patterns.
Algorithmic *Happens when models are trained on biased or unrepresentative data*, *leading to skewed predictions*. *This can happen during data collection*, *where populations might be misrepresented*, *or in labeling*, *where human biases influence outcomes*, *especially if annotators don’t reflect the broader population*. *Additionally*, *some algorithms function as black boxes*, *making it difficult to identify and correct these biases* [80,88].	Logistic regression assuming linearity, overfitting, or amplifying biases from limited samples **Mitigation:** *A priori*—Use robust, unbiased sampling strategies; define model parameters carefully; validate models with diverse datasets. *A posteriori*—Conduct sensitivity analyses; apply external validation; continuous model assessment and evaluation.	Machine learning models often struggle with biased data, which can result in underrepresenting smaller groups or amplifying the influence of larger groups. Black box algorithms can worsen this issue, making it harder to detect and correct biases (e.g., amplifying vaccination attitudes of middle-aged users while overlooking ethnic minorities). **Mitigation:** *A priori*—Diverse, representative data through open collaboration; follow established guidelines; benchmarking against health data sets (e.g., CDC surveys). *A posteriori*—Cross-validation with independent data sets; transparency; retraining with balanced data; validation against gold standard sources; supplementation with traditional data sets.	Less relevant in classical epidemiology due to simpler, scrutinizable models, making bias detection easier. When it occurs, it often derives from model assumptions. Digital epidemiology faces greater challenges with complex, black box algorithms and unstructured data, requiring advanced transparency and validation methods. LLMs (Large Language Models) may help identifying unexpected trends or anomalies by incorporating contextual knowledge.

## Digital Epidemiology in Transition

### Digital Epidemiology before the COVID-19 pandemic

The potential of Digital Epidemiology to enhance surveillance by making it faster, cheaper, and broader is not new. A notable example is the creation of a network of sentinel doctor digital records in France in 1984, focusing on influenza syndromic monitoring [11]. In this case, the network was selected to be representative, trying to correct for possible biases both *a priori* and *a posteriori*.

This is in clear contrast with Google Flu Trends (GFT), launched in 2008, which aimed to correlate online searches for flu-like symptoms with actual cases [12]. Here, the sample was composed of “people who did searches on Google,” with the search-terms ultimately being selected by Google’s researchers. While initially successful, GFT missed the 2009 pandemic onset and overestimated cases by 140% during the 2012–13 flu season [13]. It has been argued that its accuracy was compromised by spurious correlations, overfitting, and media coverage [14], issues that are also relevant when analyzing social media posts [15]. However, the first cases of the 2009 flu pandemic were reported by local news media in Mexico before being detected by the CDC or the WHO, supporting the use of nontraditional data in different languages [16]. In 2013, the CDC announced FluSight, a competition to improve flu monitoring, with the winning methodology combining “traditional” and “less traditional” data sources, such as weather and online searches [17,18].

Other efforts have leveraged online platforms to include citizen participation and self-reporting. For instance, InfluenzaNet collects flu-like symptoms from voluntary participants across 10 European countries, often anticipating official outbreak announcements [19], and similar successful approaches have been adopted in various places [20–22]. While self-reporting provides a cost-effective and ethical way to gather longitudinal data, it is subject to several sampling biases (e.g., selection, attrition) that must be corrected a posterior in implemented models (see S1 Table). For a more comprehensive comparison of digital surveillance systems and their evolution before and after the COVID-19 pandemic, see S1 Table in the Supporting information.

In the early 2000s, several countries also introduced free or low-cost phone services to offer clinical advice and triage callers. Using patient data and symptoms as inputs, these systems combine predetermined computational algorithms with curation by trained clinicians. As covering increasingly large demographics, they can be used for large-scale surveillance and have shown potential for fast outbreak detection [23].

Similarly, tracing tools based on cell phones, WiFi, or Bluetooth badges [24–26] demonstrated significant potential in spatial disease models, aiding public health interventions during outbreaks of Ebola [27], Dengue [28], Zika [29], and others [30]. However, these studies tend to oversample well-off populations [31], and even cell phone ownership alone does not guarantee access. Despite sharing some of the same limitations in sampling, approaches that use airline traffic, commuter trajectories [32], general mobility [33], or urban lights [34], have also been successful in explaining patterns of disease transmission, exemplifying the potential for post hoc bias corrections.

**Fig 2:**
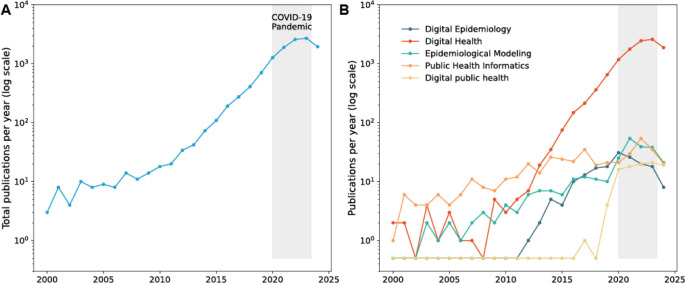
Digital Epidemiology publications over time (log scale). (**A**) Yearly total number of publications related to digital epidemiology, obtained through systematic data extraction using the PubMed API, shows a sharp increase in research activity that peaked during the COVID-19 pandemic (shaded area). (**B**) Publication trends within specific Digital Epidemiology-related domains from 2000 to 2024, illustrating the evolution of research focus across these areas.

Overall, Digital Epidemiology studies on communicable diseases steadily increased from 2005 to January 2020 (see Fig 2), leveraging text data from social media (Twitter, Facebook, and Instagram), search engines (Google and Baidu), news media, and Wikipedia. These studies aimed to predict disease dynamics, explore seasonal patterns, analyze information-seeking behavior, and detect outbreaks or pandemics, with a significant focus on the flu. They addressed both seasonal (33%) and epidemic (45%) occurrences, showcasing tools for predicting transmission pattern changes [35].

Despite this progress, there are limited instances of widespread adoption of Digital Epidemiology tools and results by public health officials, often due to a lack of validation, funding, or perceived usefulness [35]. Other contributing factors include inadequacies in digital infrastructure, the absence of a comprehensive strategy, outdated software, and a lack of interoperability between systems [36]. Rare exceptions include studies that demonstrate the value of integrating traditional and nontraditional data sources, such as the aforementioned InfluenzaNet [37], and the Guardians of Health platform, which applied crowdsourced data during the Rio 2016 Olympic Games [38].

Early pioneering initiatives in this field also include the Program for Monitoring Emerging Diseases (ProMED) [39] and HealthMap [40]. In fact, before 2020, WHO and independent initiatives routinely tracked various diseases (e.g., Zika fever, Ebola, Dengue), identifying around 3,000 potential outbreak signals monthly [41].

In summary, by early 2020, these systems showed promise but lacked sufficient support. For instance, the Global Public Health Intelligence Network (GPHIN), credited with early detection of MERS, Ebola, and the 2009 flu pandemic, was halted in 2019 [42]. As a result, none of these tools could prevent the spread of SARS-CoV-2, leading to the second pandemic of this century.

### The impact of the COVID-19 pandemic

At the beginning of 2020, a surge in hospitalizations and deaths was traced to infections from a new coronavirus and led to strong and worldwide government-enforced containment measures. These circumstances spurred massive efforts in data collection, aiming to track infections and understand the various dimensions of the pandemic dynamic. Countries were expected to produce detailed case reports, platforms collaborated to share data and develop contact tracing apps, and lockdowns shifted communication online, resulting in an abundance of medically relevant data.

As illustrated in Fig 2, Digital Epidemiology research peaked during the pandemic, possibly due to (1) an increase in both the quantity and quality of data; (2) the emergence of a new data-sharing culture; and (3) a significant push towards technological solutions, as discussed in [9] and further evidenced by other social transformations [43].

However, the pandemic also highlighted several challenges. Many data sets, although advantageous, were statistically unrepresentative and contained biases that could not be corrected *a priori*. For example, the widely used data set from Johns Hopkins University Center for Systems Science and Engineering (JHU CSSE) [44] provided detailed information on positive cases, mortality, and ICU occupancy, facilitating model development and predictions. Yet, the lack of standardized testing across countries hindered the reliability of official numbers and complicated comparisons, especially given the varying national policies on testing and classification of COVID-19–related deaths [45]. For instance, a study examining testing rates in Southeast Asia demonstrated how these were influenced by economic capacity, healthcare investment, and governance, resulting in varied detection rates across countries [46]. Similarly, many COVID-19 prediction models that were developed rapidly during the pandemic suffered from a high risk of bias, overfitting, and inadequate validation. These challenges further complicated the use of such models in clinical settings [47]. These inconsistencies not only distorted the perceived severity of outbreaks but also affected the evaluation of public health measures.

Another important shift was in the use of digital tools and wearable devices. Sales of pulse oxymeters and heart-rate monitors surged [48], and digital contact tracing apps gained prominence [49,50]. Despite the potential of the latter to reduce exposure and transmission [51], these apps faced significant challenges that exemplify the challenges of Digital Epidemiology in general. First, the collaboration between Google and Apple, to create an opt-in smartphone-based system [52,53], revealed the difficult balance between efficiency and privacy, particularly when a flaw in the Android system compromised anonymity [54]. Second, insufficient collaboration between public health experts, practitioners, and app developers, led to implementation problems [55,56]. Third, limited and biased adoption (possibly due to mistrust, dysfunction, and unequal access to smartphones) made false-negatives harder to correct and might have created a false sense of safety within some groups.

The pandemic also gave rise to the widespread use of mobile-based open-source mobility data from platforms such as Facebook, Google, and Apple. These data sets were instrumental in understanding the effect of changes in mobility, the pathogen’s evolution, mobility-based inequalities and in minimizing disease spread [57–60]. However, as mentoned before, these data sets often exclude older and poorer communities and lower socioeconomic groups were less able to comply with lockdown measures [60,61].

These sampling biases are particularly important when this data is used to train machine learning algorithms, which were extensively used for now- and fore-casting. Due to their nature, discrepancies and class imbalances in training data sets are perpetuated and might even be amplified, leading to faulty predictions. Naturally, *a posteriori* corrections of both data sets and models are possible, but far from trivial. Furthermore, the rapidly changing situation meant that data used for training models could quickly become outdated, failing to account for evolving factors such as viral mutations, changes in public behavior, and the rollout of vaccines. This required continuous updating and retraining of models to ensure their relevance, which was both resource-intensive and difficult to implement effectively.

One of the potentially long-lasting impacts of the pandemic has been the sharp increase in telemedicine and chatbot usage. These services grew more than 20-fold in the US shortly after the pandemic began [62], with the goal of limiting pathogen exposure and reduce in-person visits. Examples include using telemedicine in chronic disease patient management [63] or offering remote provider-to-provider specialist guidance [64]. Mobile applications were developed for self-triage, self-scheduling, and delivering health information [65], and tools like HealthBuddy+, developed by WHO and UNICEF, exemplify how digital platforms can provide reliable information and monitor public sentiment during health crises [66]. These and other digital health innovations will likely continue to evolve and become integral to everyday healthcare, but sustaining these tools requires significant investment and restructuring.

In fact, the observed peak in Digital Epidemiology research (Fig 2) was followed by a post-pandemic decline in publication rates. This could be due to several factors and we list 3: (1) a shift in research focus as the immediate crisis stabilized, leading to renewed attention to other scientific areas; (2) research fatigue around the topic; and (3) data scarcity, where access to previously available data sets became limited due to reduced testing, decreased data sharing by public health agencies, and restrictions on access to private data, including social media data from platforms like Twitter and Facebook, and the discontinuation of resources such as Google Mobility Reports.

In summary, despite the significant potential and growing influence of Digital Epidemiology to revolutionize public health responses, existing solutions were not yet sufficiently developed to achieve a large-scale impact. The following section will address some of the most pressing challenges in Digital Epidemiology, particularly those related to generalizability, representativeness, and practical implementation.

### Strengths and challenges in Digital Epidemiology

There is a persistent idea that online data and digital data sources are irremediably flawed when compared with Classical Epidemiology studies, due to concerns such as bias, privacy issues, and ethical challenges [67,68]. However, it is increasingly clear that both systems have strengths and weaknesses, and that Digital Epidemiology can unveil fundamental health-related aspects that traditional methodologies do not provide. We have argued that, while both fields employ *a priori* and *a posteriori* methods to address biases, Classical Epidemiology typically integrates bias prevention into the study design from the outset. In contrast, the unstructured and secondary nature of data collection in Digital Epidemiology requires a greater dependence on *a posteriori* corrections.

Table 1 highlights biases and statistical challenges common to both Classical and Digital Epidemiology, focusing on those that best illustrate the differences (and complementarity) between the 2 approaches. For example, while traditional studies often suffer from limited data (in numbers, space, and time), digital epidemiology models suffer from a lack of validation (and, in the case of overfitting, possibly even an excess of data). While no data or study is ever perfect or complete [69], it should be recognized that methods to minimize bias in traditional studies are better established and that current post-collection correction is particularly challenging for 3 main reasons: (1) the biases present in sources like social media, mobile apps, or online searches are difficult to anticipate (e.g., platform-user demographics might not be known and user-generated content might reflect unknown and fast-changing cultural or social biases); (2) given the size of the data and the sensitivity of many current computational models, the carried biases may be easily amplified, masquerading as signal; (3) many machine learning models rely on proxy data, and the choice of proxies introduces biases as well [70,71]. Moreover, many of the mitigation strategies presented in the table are often beyond the direct control of researchers, since they often depend on the willingness of data creators, owners, and handlers, such as social media platforms or government entities responsible for data storage. In fact, some key studies on mental health contagion were only possible with the involvement of the platforms themselves [72], which may or may not be open to academic collaboration. Therefore, while combining the strengths of classical methods with the agility and scope of digital approaches is key [73], it is equally important to recognize and address these dependencies and limitations to ensure more effective and equitable use of digital epidemiological methods. We note that the list and classification presented here are neither exhaustive nor rigid, and they are not mutually exclusive. However, we find that by focusing on the statistical challenges of addressing biases, we can more effectively identify gaps and allocate resources.

### Recommendations and future prospects

The integration of Digital Epidemiology with Classical Epidemiology holds enormous potential for advancing public health research, and it is crucial to define strategies that can minimize bias and prioritize transparency, inclusion, and the ethical application of digital tools.

Below, we present a set of recommendations, aimed at fostering a more balanced, transparent, and evidence-based approach to this transition.

- **Complementarity and validation**: While digital data streams offer real-time spatial and temporal insights that can help design further epidemiological studies [29], these should be combined with traditional methods to validate digital data sets, especially for populations with limited digital access. This is crucial for interpretability and generalizability, particularly of black box models.
- **Data availability and structure**: Establish international standards for data collection, analysis, and sharing, ensuring metadata transparency and alignment with the FAIR framework. Governments should provide open access to relevant data sets. Both the recently announced European Health Data Space (EHDS) [89], and the new directives from the European Center for Disease Control (ECDC)—case based versus event based—include important guidelines for such standardization and cross-border sharing by requiring member states to establish a common framework for data access, interoperability, and patient privacy [90]. These standards should also include clear ways to highlight possible biases in collection, curation, and others.
- **Collaboration with private sector**: Engage private companies (social media, hospitals, tech firms) as long-term partners in public health preparedness. Foster data-sharing practices to develop accessible, sustainable health tools beyond emergency contexts. In parallel, be alert to the biases introduced by conflicts of interest, particularly during research and policy implementation [91]. This also aligns with the EHDS [89], which offers a timely opportunity to merge Digital and Classical Epidemiology techniques.
- **Disease identification**: Apply AI-driven multimodal tools to improve disease classification. Integrating diverse data layers—genomics, imaging, and health records—can enhance disease prediction [92]. Examples may include using natural language processing tools combined with medical imaging data to identify syndromic differences more accurately or leveraging electronic prescriptions, combined with patient demographics and clinical imaging data, to infer diagnosis. Generative AI can also further address data imbalances, improving identification accuracy. In all cases, balancing predictive accuracy with causal decision-making is crucial for real-world application [93].
- **Digital adaptation**: Address the digital health paradox, where the groups that could most benefit from free and fast digital tools often face the greatest barriers, as seen during the COVID-19 pandemic, when underprivileged communities were hit harder. It is essential to engage representatives from different communities when developing telemedicine and digital health tools to ensure that they are accessible and effective [94]. Further research on existing barriers (technological, sociological, operational) and effectiveness should be prioritized.
- **Public participation**: Encourage participatory data collection to enhance data literacy, empower communities, and address imbalances in epidemiological studies conducted by the Global North on the Global South [69,95,96].
- **Research and implementation**: Strengthen collaboration between academia, tech providers, and public health institutions. A multidisciplinary approach is crucial: experts from public health, data science, ethics, sociology, and clinical medicine, among others, should collaborate to refine and validate findings. Again, the EHDS might prove central in promoting such collaboration, cross-border research, and data sharing [89]. Prioritize research on AI fairness, algorithmic bias, transparency, and explainability to address biases. Facilitate the adoption of effective tools through peer-reviewed publications and programs, ensuring fairness and transparency.
- **Communication and disinformation**: Promote transparent data sharing while preventing and mitigating misinformation with multidisciplinary collaboration. Establish databases for news stories and rumors to ensure effective communication strategies [97]. Apply multidimensional data visualization to identify gaps and patterns, and incorporate behavioral sciences to better interpret diverse data streams and outputs, ensuring more accurate and impactful communication.
- **Transparency**: Emphasize transparency in both developing and implementing digital solutions. Use glass box models over black box methods for better understanding and accountability. Engage stakeholders, including public health officials, patients, and community representatives, to review models, preventing model drifting through diverse input. Maintain version control for algorithms, clearly documenting changes. During implementation, ensure data sources, methodologies, and regular audit findings are openly shared to facilitate bias detection and correction, building trust and effectiveness in digital health.
- **Performance assessment**: An iterative refinement process is essential, where models are continuously tested, evaluated, and improved using explainable methods to identify potential biases and inaccuracies early on. Implementing background performance assessments in real-world settings, especially outside critical moments such as disease outbreaks, ensures digital tools are effective, safe, and equitable before widespread deployment.

## Discussion

In this paper, we discussed how the desired joint implementation of Classical and Digital Epidemiology implies that data sets designed with *a priori* statistical rigor (e.g., epidemiological surveys, census, weather) are combined with systems designed for different goals (e.g., insurance claims, telephone helplines, social media posts) that require *a posteriori* debiasing.

We further argued that key steps are necessary to advance this transition: (1) recognizing that classical Epidemiology and Digital Epidemiology can validate and improve each other; (2) acknowledging that addressing biases is not a one-size-fits-all method, demanding not only different statistical and technical solutions but also engagement with various communities and stakeholders; and (3) establishing multidisciplinary approaches and infrastructures. We briefly describe them below.

First, bias and complementarity are evident in the use of online data and machine-learning models for disease monitoring. An essential concern arises from the amplification of existing inequalities, where underrepresented groups in data sets are also underrepresented in the analysis—applicable to both Digital Epidemiology and Classical Epidemiology data sets. The term “amplify” carries 2 meanings: first, these tools may exacerbate social divides, and second, they highlight these inequalities, making them more visible. While biases in the data mirror real-world disparities, increased visibility might aid in addressing these issues more effectively. However, current debiasing approaches (in both Classical Epidemiology and Digital Epidemiology, whether they correct data sets or methods) rely on already knowing what the putative biases are: unknown biases remain uncorrected and, with the current tools, incorrigible. This highlights not only the importance of including many demographics and cross-validating tools, but also the need for more research on bias correction methods.

Similarly, the challenge of discerning signal from noise [98] offers another example of the importance of multimodal approaches: as different motivations drive online searches or social media posts (as made evident in the GFT case) identifying the relevant signal is crucial and we need behavioral or other models, capable of distinguishing between searches driven by actual cases and those influenced by media or fear.

In all these situations, collaboration between different fields of knowledge plays a fundamental role. Such synergies require long-term projects focused on data quality [99] and on designing testing strategies that prioritize validation and safe implementation rather than just predictions or therapeutics. For example, advanced analytical tools, such as machine learning and multimodal AI, can help refine case definitions in Digital Epidemiology by integrating diverse data inputs, enhancing the accuracy and timeliness of public health responses. But while this approach shows promise, predictive models often rely on correlations, which may not always translate into actionable decisions at the individual level. In an extreme example, while predicting that social distancing effectively reduces contagion, such models tell us little about how this distancing should be implemented at a granular level, such as when, where, and for whom it would be most effective. Appropriate interventions and decisions require a deeper causal understanding of agents and situations. Moreover, these models are blind to ethical risks and the potential for exacerbating inequalities must be carefully managed, especially in regions with less digital access or strong governance.

In fact, despite it being commonplace to mention that the epidemiology of infectious diseases is a complex system, strategies are rarely designed to include integration between data, models, ecosystems, and response.

Which brings us to the last point of infrastructure building. Similar to what happens with weather prediction, there is great promise in the creation of large multidisciplinary institutions (WHO’s Hub for Pandemic and Epidemic Intelligence, UK’s Center for Pandemic Preparedness, the National Center for Epidemic Forecasting and Outbreak Analytics, or the Rockefeller Pandemic Prevention Institute, in the USA) that could also help guide policymakers to implement targeted measures. In Europe, several COVID-19 forecasting and scenario modeling hubs were established during the pandemic crises and, more recently, the ECDC created 2 respiratory diseases hubs for forecasting and scenario modeling [100,101]. From a global perspective, the WHO also announced support for regional hubs for pandemic and epidemic intelligence.

However, despite these promising initiatives, many regions, such as South East Asia and Africa [102], still lack such institutions, and there are concerns that the momentum to drive these efforts forward might be diminishing.

Naturally, such approaches require not only adequate infrastructures, but also clear standards, data sharing at local and global scales, and significant funds. If not now, “when the next major crisis is on our doorstep, we’re not going to be any more prepared to respond to it than we were with this last one” [103]. It is imperative that the lessons learned from COVID-19 drive us toward a more resilient, integrated, and forward-thinking approach to epidemiology.

## Supporting information

S1 Appendix Definitions. In the Supplementary Material, a comprehensive list of definitions is provided to clarify and standardize the terminology used throughout this paper. Key public health and epidemiological terms such as ‘Public Health’, ‘Epidemiology’, ‘Digital epidemiology’, ‘Epidemic intelligence’, ‘Surveillance’, ‘Tele-medicine’, ‘Fore-casting’, and ‘Now-casting’ are defined to ensure a common understanding.
(PDF)

S1 Table Digital surveillance systems overview. Presents a summary of the evolution of digital surveillance systems for infectious diseases, highlighting the significant changes from pre-COVID-19 methods to adaptations during the pandemic and future perspectives. It covers areas such as syndromic and lab surveillance, contact tracing, digital medicine, spatial analysis, and communication strategies, emphasizing the technological advancements, challenges, and potential improvements for public health management in a post-pandemic world.
(PDF)
