# Supplementary material for: Epidemiological methods in transition: Minimizing biases in classical and digital approaches
Source: PLOS Digit Health. 2025 Jan 13;4(1):e0000670. doi: 10.1371/journal.pdig.0000670 (PMC11730375; doi:10.1371/journal.pdig.0000670)
Supplement: S1 Appendix — In the Supplementary Material, a comprehensive list of definitions is provided to clarify and standardize the terminology used throughout this paper. Key public health and epidemiological terms such as ‘Public Health’, ‘Epidemiology’, ‘Digital epidemiology’, ‘Epidemic intelligence’, ‘Surveillance’, ‘Tele-medicine’, ‘Fore-casting’, and ‘Now-casting’ are defined to ensure a common understanding. (PDF) [file pdig.0000670.s001.pdf]

Supplementary Material

Definitions

Public Health - science and practice of preventing disease, prolonging life and promoting physical and mental health and well-being [1]

Epidemiology - study of how disease is distributed in populations and the factors that influence or determine this distribution [2]

Digital epidemiology - originally defined as use of digital data collected for non-epidemiological purposes in epidemiological studies [3], we offer the alternative use of data collected without *a priori* concerns of statistical representativeness in epidemiological studies

Epidemic intelligence - all activities related to early identification of potential health hazards, their verification, assessment and investigation aiming to recommend public health control measures [4]

Surveillance - collection, collation, and analysis of data and the dissemination to those who need to know so that an action can result [5].

Tele-medicine - technology-mediated connection between providers and patients in different locations, allowing for diagnosis, monitoring, triage, patient follow-ups, etc. [6].

Fore-casting - predicting future events based on a foreknowledge acquired and implies planning under conditions of uncertainty [7].

Now-casting - prediction of the present, the very near future and the very recent past [8].

Bias - refers to a systematic deviation in a study's design or execution that leads to errors. This deviation arises from issues in how participants are selected or in the methods used to collect data on exposure and/or disease. As a result, the findings from the study are likely to differ from the actual, underlying truth.

References

1. C-EA Winslow, “The untilled fields of public health,” *Science*, vol. 51, no. 1306, pp. 23–33, 1920, American Association for the Advancement of Science.

2. Gordis. *Epidemiology*. Elsevier/Saunders, 2009.

3. M. Salathe, L. Bengtsson, T. J. Bodnar, D. D. Brewer, J. S. Brownstein, C. Buckee, E. M. Campbell, C. Cattuto, S. Khandelwal, P. L. Mabry, et al., “Digital epidemiology,” 2012, Public Library of Science San Francisco, USA.

4. C. Paquet, D. Coulombier, R. Kaiser, M. Ciotti, “Epidemic intelligence: A new framework for strengthening disease surveillance in Europe,” Eurosurveillance, vol. 11, no. 12, pp. 5–6, 2006, European Centre for Disease Prevention and Control.

5. S. B. Thacker, K. Choi, P. S. Brachman, “The surveillance of infectious diseases,” JAMA, vol. 249, no. 9, pp. 1181–1185, 1983, American Medical Association.

6. A. S. Kazley, A. C. McLeod, K. A. Wager, “Telemedicine in an international context: definition, use, and future,” in *Health Information Technology in the International Context*, vol. 12, pp. 143–169, 2012, Emerald Group Publishing Limited.

7. S. A. Lauer, A. C. Brown, N. G. Reich, “Infectious Disease Forecasting for,” in *Population Biology of Vector-Borne Diseases*, p. 45, 2020, Oxford University Press.

8. D. Giannone, L. Reichlin, and D. Small, “Nowcasting: The real-time informational content of macroeconomic data,” *Journal of Monetary Economics*, vol. 55, no. 4, pp. 665–676, 2008, Elsevier.
