# Supplementary material for: Epidemiological methods in transition: Minimizing biases in classical and digital approaches
Source: PLOS Digit Health. 2025 Jan 13;4(1):e0000670. doi: 10.1371/journal.pdig.0000670 (PMC11730375; doi:10.1371/journal.pdig.0000670)
Supplement: S1 Table — Presents a summary of the evolution of digital surveillance systems for infectious diseases, highlighting the significant changes from pre-COVID-19 methods to adaptations during the pandemic and future perspectives. It covers areas such as syndromic and lab surveillance, contact tracing, digital medicine, spatial analysis, and communication strategies, emphasizing the technological advancements, challenges, and potential improvements for public health management in a post-pandemic world. (PDF) [file pdig.0000670.s002.pdf]

Digital surveillance systems overview

Table 1. Digital surveillance systems overview (continued)

		Pre-COVID	Post-COVID-19	Future directions	Some References
**Syndromic**	Surveillance	**Clinical:** Mostly in-person symptoms collection; Sentinel Doctors; Inability to detect outbreaks in real-time; **Online:** Efforts to analyse social media posts and online searches (e.g. Google Flu Trends); Participatory syndromic surveillance (e.g. InfluenzaNet)	**Clinical:** Semi-automatic syndromic-based triage; Tele-medicine; **Online:** Increased participatory syndromic surveillance; Very intense media reporting affecting datasets; Hard to discriminate symptoms of different respiratory viruses; Tools tested in large scale with promising results; Fragmented approaches	Multi-system approach: self-reporting, social media & traditional methods; systematic testing of different respiratory viruses for validation; improve controls to/and reduce sampling bias	[1], [4], [2], [3]
**Lab Surveillance**	**Testing**	Lab testing (mainly PCR) of known pathogens; Testing of symptomatic patients or when therapeutic was in doubt; Sentinel Doctors; Highly trained staff and facilities; Poor integration with electronic medical records; Significant delays between sampling, testing and result sharing	First pandemic with population-scale screening available; point-of-care (POC) widespread tests and part of the routine (home, local clinic, workplace, etc.); collaboration across commercial, clinical, government, and research organizations; Increased genomic surveilance; cost & effectiveness of mass testing still debated	Adoption of wearables; Novel low-cost methods (molecular, AI/Machine Learning inference); Less invasive sampling techniques (e.g., saliva, cough, breathing & voice); reinforce communication on testing techniques limitations; simultaneous and automated reporting of test results to patients and clinical & public-health systems	[5], [6], [3]

	**Wastewater surveil-lance**	History of tracking infectious diseases (e.g. poliovirus), antibiotic use and drug consumption; not integrated with other data, tools and reporting systems (incidence, hospitalizations, etc.)	Used to predict community surges but integration with reporting systems still limited; variants of concern (VOC) identified weeks prior to clinical samples; lack of standardization; lack of granularity (cannot identify outbreak location when area served is too wide)	Expand to other pathogens; invest in standardization; integrate with environmental and veterinary monitoring system to identify diseases with zoonotic potential	[7–12]
**Contact Tracing**		**Manual:** Well documented method, mainly manual; Sensitive to: community trust, privacy concerns, possible political interference, availability of trained professionals, incomplete databases; **Digital:** Initial efforts to use digital badges and other technological tools	**Manual:** traditional method kept with human resource limitations; **Digital:** Deployment of cell-phone based apps; Google & Apple collaboration; Effectiveness, ethical considerations, security, technical issues raised	Improve implementation (technological, social, ethical); Ensure transparency; Include communities and multiple stakeholders; take human behavior into consideration	[13], [14], [15], [16]

**Digital Medicine**	**Wearables/apps:** medical apps started being prescribed by physicians (e.g. for glucose monitoring); lack of evaluation in Randomized Controlled Trials; low quality of the evidence; **Tele-medicine:** Online and on-phone triage systems; increased number of digital medical records and health-related platforms; **Sensors:** implemented mostly inside hospitals and clinics to measure vital signs	**Wearables/apps:** preliminary results for SARS-CoV-2 infection detection through heart rate variability, oxygen saturation and respiration rate; validation and accuracy to be determined; **Tele-medicine:** digital health solutions became a necessity (tele-medicine, triage for SARS-Cov2 testing or isolation); absence or insuficient regulation; uneven adoption and access: higher-income earners, highly educated, and with chronic conditions being the likeliest adopters	More robust RCTs; invest in coherent and accessible data infrastructures; invest in digital transformation in clinical contexts; support citizen access to internet and mobile interfaces; invest in education and digital literacy; invest in secure and privacy protecting platforms.	[17], [18], [19]

**Spacial Analysis**	**Mobility**	Mobile phone data used to trace movement across large numbers of individuals; Combined with other information (climatic, vector information, demographics) improved disease spreading predictions (e.g., 2011 dengue in Pakistan, 2017 chikungunya in Bangladesh)	Facebook's Data-For-Good, Google's COVID-19 Community Mobility Reports; Apple's COVID-19 mobility datasets; Control measures adapted after inequities were revealed through mobility analysis; phylogeographic component of SARS-CoV-2 genomic reconstruction informed by Google mobility data; limited metadata; no long-term commitment by companies to share data	Evaluate implemented mobility-control policies (timing, effectiveness, and stringency); enrich mobility source data to ensure data accuracy and less dependency on providers; improve data ownership by citizens and consider the need for informed consent prior to sharing (even when aggregated)	[20], [21], [22], [23], [24], [25]
	**Geographical Information Systems (GIS)**	Widely used in resource-constrained settings and countries with high burden of infectious diseases; Technological developments and availability of GIS allowed advances	Used to manage lockdowns, detect outbreaks, and clusters of infection; main sources of data: contact tracing data, cell-phones (geolocation, payments, points of connection), flights or social media, socio-economic characteristics of the population and its urban structure, polls, participatory GIS and satellite images; web map viewers highly used to inform public and specialists	Exploring the different dimensions and regional patterns of social determinants of health	[26]

**Communication & Data Visualization**	**Data Visualization:** Easy access to data visualization software tools; rising popularity of data journalism; easy image sharing on social media	**Communication:** very rigid and formal communication channels between public health authorities and the general public	**Data Visualization:** Widespread use of dashboards to monitor (disease, resources) and communicate with policy makers, scientists, healthcare providers, general public; data used mainly from traditional & official sources; can be used to expose inequities and as bait to spread disinformation; **Communication:** poor communication skills from many researchers and public officials	Further evaluate how data visualizations influence risk perceptions to improve pandemic communication; use it as a tool to improve the population digital literacy; use it as a validation tool within the target communities of the analysis; research impact(s) and possible mitigation roles on disinformation spread	[27–30]

References

1. M. S. Smolinski, A. W. Crawley, K. Baltrusaitis, R. Chunara, J. M. Olsen, O. Wójcik, M. Santillana, A. Nguyen, and J. S. Brownstein, "Flu near you: Crowdsourced symptom reporting spanning 2 influenza seasons," *American Journal of Public Health*, vol. 105, no. 10, pp. 2124–2130, 2015.

2. A. S. Maharaj, J. Parker, J. P. Hopkins, E. Gournis, I. I. Bogoch, B. Rader, C. M. Astley, N. Ivers, J. B. Hawkins, N. VanStone, et al., "The effect of seasonal respiratory virus transmission on syndromic surveillance for COVID-19 in Ontario, Canada," *The Lancet Infectious Diseases*, vol. 21, no. 5, pp. 593–594, 2021.

3. J. Budd, B. S. Miller, E. M. Manning, V. Lampos, M. Zhuang, M. Edelstein, G. Rees, V. C. Emery, M. M. Stevens, N. Keegan, et al., "Digital technologies in the public-health response to COVID-19," *Nature Medicine*, vol. 26, no. 8, pp. 1183–1192, 2020.

4. M. R. Desjardins, ”Syndromic surveillance of COVID-19 using crowdsourced data,” *The Lancet Regional Health–Western Pacific*, vol. 4, 2020.

5. J. Han, T. Xia, D. Spathis, E. Bondareva, C. Brown, J. Chauhan, T. Dang, A. Grammenos, A. Hasthanasombat, A. Floto, et al., ”Sounds of COVID-19: exploring realistic performance of audio-based digital testing,” *NPJ Digital Medicine*, vol. 5, no. 1, pp. 1–9, 2022.

6. N. K. Tran, S. Albahra, H. Rashidi, L. May, ”Innovations in infectious disease testing: Leveraging COVID-19 pandemic technologies for the future,” *Clinical Biochemistry*, 2022.

7. A. Bivins, D. North, A. Ahmad, W. Ahmed, E. Alm, F. Been, P. Bhattacharya, L. Bijlsma, A. B. Boehm, J. Brown, et al., ”Wastewater-based epidemiology: global collaborative to maximize contributions in the fight against COVID-19,” 2020, ACS Publications.

8. N. Fahrenfeld and K. J. Bisceglia, ”Emerging investigators series: Sewer surveillance for monitoring antibiotic use and prevalence of antibiotic resistance: Urban sewer epidemiology,” *Environmental Science: Water Research & Technology*, vol. 2, no. 5, pp. 788–799, 2016.

9. S. Castiglioni, I. Senta, A. Borsotti, E. Davoli, and E. Zuccato, ”A novel approach for monitoring tobacco use in local communities by wastewater analysis,” *Tobacco Control*, vol. 24, no. 1, pp. 38–42, 2015.

10. S. L. Servetas, K. H. Parratt, N. E. Brinkman, O. C. Shanks, T. Smith, P. J. Mattson, and N. J. Lin, ”Standards to support an enduring capability in wastewater surveillance for public health: Where are we?”, *Case Studies in Chemical and Environmental Engineering*, vol. 6, 100247, 2022.

11. P. Kilaru, D. Hill, K. Anderson, M. B. Collins, H. Green, B. L. Kmush, and D. A. Larsen, ”Wastewater surveillance for infectious disease: a systematic review”, *MedRxiv*, 2021.

12. B. M. Gomes, C. B. Rebelo, and L. A. de Sousa, ”Public health, surveillance systems and preventive medicine in an interconnected world,” in *One Health*, pp. 33–71, Elsevier, 2022.

13. J. Müller and M. Kretzschmar, ”Contact tracing–Old models and new challenges,” *Infectious Disease Modelling*, vol. 6, pp. 222–231, 2021, Elsevier.

14. O. O. Olu, M. Lamunu, M. Nanyunja, F. Dafae, T. Samba, N. Sempiira, F. Kuti-George, F. Z. Abebe, B. Sensasi, A. Chimbaru, et al., ”Contact tracing during an outbreak of Ebola virus disease in the Western area districts of Sierra

Leone: lessons for future Ebola outbreak response," *Frontiers in public health*, vol. 4, p. 130, 2016, Frontiers Media SA.

15. A. Akinbi, M. Forshaw, V. Blinkhorn, "Contact tracing apps for the COVID-19 pandemic: a systematic literature review of challenges and future directions for neo-liberal societies," *Health Information Science and Systems*, vol. 9, no. 1, pp. 1–15, 2021, Springer.

16. L. O. Danquah, N. Hasham, M. MacFarlane, F. E. Conteh, F. Momoh, A. A. Tedesco, A. Jambai, D. A. Ross, H. A. Weiss, "Use of a mobile application for Ebola contact tracing and monitoring in northern Sierra Leone: a proof-of-concept study," *BMC Infectious Diseases*, vol. 19, no. 1, pp. 1–12, 2019, BioMed Central.

17. O. Byambasuren, S. Sanders, E. Beller, P. Glasziou, "Prescribable mHealth apps identified from an overview of systematic reviews," *NPJ Digital Medicine*, vol. 1, no. 1, pp. 1–12, 2018, Nature Publishing Group.

18. J. D. Iqbal and N. Biller-Andorno, "The regulatory gap in digital health and alternative pathways to bridge it," *Health Policy and Technology*, vol. 11, no. 3, pp. 100663, 2022, Elsevier.

19. J. DeSilva, R. Prensky-Pomeranz, and M. Zweig, "Digital Health Consumer Adoption Report: How COVID-19 accelerated digital health beyond its years. Stanford Center for Digital Health & Rock Health," 2021. [Online; accessed 01-December-2022]. Available: `https://rockhealth.com/insights/digital-health-consumer-adoption-report-2020/`.

20. A. Wesolowski, C. O. Buckee, K. Engø-Monsen, and C. J. E. Metcalf, "Connecting mobility to infectious diseases: the promise and limits of mobile phone data," *The Journal of infectious diseases*, vol. 214, no. suppl_4, pp. S414–S420, 2016.

21. R. E. Baker, A. S. Mahmud, I. F. Miller, M. Rajeev, F. Rasambainarivo, B. L. Rice, S. Takahashi, A. J. Tatem, C. E. Wagner, L.-F. Wang, et al., "Infectious disease in an era of global change," *Nature Reviews Microbiology*, vol. 20, no. 4, pp. 193–205, 2022.

22. A. Wesolowski, T. Qureshi, M. F. Boni, P. R. Sundsøy, M. A. Johansson, S. B. Rasheed, K. Engø-Monsen, and C. O. Buckee, "Impact of human mobility on the emergence of dengue epidemics in Pakistan," *Proceedings of the National Academy of Sciences*, vol. 112, no. 38, pp. 11887–11892, 2015.

23. A. S. Mahmud, M. I. Kabir, K. Engø-Monsen, S. Tahmina, B. K. Riaz, M. A. Hossain, F. Khanom, M. R. Rahman, M. K. Rahman, M. Sharmin, et al.,

"Megacities as drivers of national outbreaks: The 2017 chikungunya outbreak in Dhaka, Bangladesh," *PLoS Neglected Tropical Diseases*, vol. 15, no. 2, e0009106, 2021.

24. S. Chang, E. Pierson, P. W. Koh, J. Gerardin, B. Redbird, D. Grusky, and J. Leskovec, "Mobility network models of COVID-19 explain inequities and inform reopening," *Nature*, vol. 589, no. 7840, pp. 82–87, 2021.

25. M. Zhang, S. Wang, T. Hu, X. Fu, X. Wang, Y. Hu, B. Halloran, Z. Li, Y. Cui, H. Liu et al., "Human mobility and COVID-19 transmission: a systematic review and future directions," *Annals of GIS*, pp. 1–14, 2022.

26. I. Franch-Pardo, B. M. Napoletano, F. Rosete-Verges, and L. Billa, "Spatial analysis and GIS in the study of COVID-19. A review," *Science of the total environment*, vol. 739, pp. 140033, 2020.

27. E. Dong, J. Ratcliff, T. D. Goyea, A. Katz, R. Lau, T. K. Ng, B. Garcia, E. Bolt, S. Prata, D. Zhang et al., "The Johns Hopkins University Center for Systems Science and Engineering COVID-19 Dashboard: data collection process, challenges faced, and lessons learned," *The Lancet Infectious Diseases*, 2022.

28. I. Boutron, A. Chaimani, J. J. Meerpohl, A. Hróbjartsson, D. Devane, G. Rada, D. Tovey, G. Grasselli, P. Ravaud, and the COVID-NMA Consortium, "The COVID-NMA project: building an evidence ecosystem for the COVID-19 pandemic," *Annals of Internal Medicine*, vol. 173, no. 12, pp. 1015–1017, 2020.

29. P. Kahn, H. Dubberly, and D. Rodighiero, "COVIC: Collecting Visualizations of COVID-19 to Outline a Space of Possibilities," *Design Issues*, vol. 38, no. 4, pp. 44–62, 2022.

30. L. Padilla, H. Hosseinpour, R. Fygenson, J. Howell, R. Chunara, and E. Bertini, "Impact of COVID-19 forecast visualizations on pandemic risk perceptions," *Scientific Reports*, vol. 12, no. 1, pp. 1–14, 2022.
